# Parkinsonism and Sjögren's Syndrome: A Fortuitous Association or a Shared Immunopathogenesis?

**DOI:** 10.1155/2015/432910

**Published:** 2015-05-31

**Authors:** Mariem Kchaou, Nadia Ben Ali, Intissar Hmida, Saloua Fray, Hela Jamoussi, Mohamed Jalleli, Slim Echebbi, Afef Achouri, Samir Belal

**Affiliations:** ^1^Neurological Department, Charles Nicolle Hospital, Boulevard du 9 Avril, Beb Souika, 1016 Tunis, Tunisia; ^2^Faculty of Medicine of Tunis, Tunisia; ^3^El Manar University, Tunisia

## Abstract

*Background*. The Sjögren Syndrome (SS) can include various manifestations of central nervous system impairment. Extrapyramidal signs are known to be very rare and unusually discovered on early onset in this pathology. *Observation*. A 46-year-old woman with a history of progressive Parkinsonism for 6 years and a normal brain magnetic resonance imaging was partially improved with levodopa therapy. The later discovery of a sicca syndrome led to performing of further investigations, which revealed the presence of anti-SSA antibodies and a sialoadenitis of grade 4 according to Chisholm's classification on labial salivary gland biopsy. The diagnosis of primary SS was established and the adjunction of corticotherapy has remarkably improved Parkinson's signs without use of other immunosuppressive agents. *Conclusion*. Based on these findings, we discuss the hypothesis of either a causal link between SS and Parkinsonism or a fortuitous association of two distinct pathologies with or without a shared immunopathogenesis.

## 1. Introduction

Primary Sjögren Syndrome (pSS) is a chronic autoimmune disease of the exocrine glands characterized by focal lymphocytic infiltration and destruction of these glands. It is a common disease with female preponderance. The main neurological impairment associated with SS is peripheral neuropathy [1]. The central nervous system involvement occurs only in 0–31% of cases [1, 2]. This frequency is probably underestimated since central signs are often precocious and initially nonassociated with systemic signs that suggest the diagnosis [2]. Only few incidental observations of extrapyramidal signs have been so far described. An exhaustive bibliographic search allows the identification of only 14 cases describing Parkinsonism associated with pSS published to date [3–12]. In these cases, direct accountability of SS was most often suggested by clinical, radiological data and treatment response [9–11]. This study reports a new case of “Parkinsonism and SS” association and discusses two hypotheses for this relation: Is there really a common anatomical basis between these two syndromes as suggested by the majority of authors? Or is it a fortuitous association of two distinct diseases?

## 2. Observation

A 46-year-old right-handed woman had rest tremor in the left upper arm for 6 years with a record of depressive syndrome. From 3 years, she developed gradually movements slowing down, the tremor reached the right upper arm, and legs were lately involved. First neurological examination in August 2013 revealed a Parkinsonian syndrome. Rest tremor was the most prominent sign. Slight muscle rigidity and bradykinesia were found in the left limbs. Hyperactive tendon reflexes were seen in four limbs without sign of Babinski reflex. She had no postural instability or gait abnormality. Physical examination was normal. Liver function and cupric tests were normal. Brain MRI T1-weighted sequences, T2 FLAIR, and T1 with gadolinium injection were also normal. The levodopa test with 125 mg was positive and improved 40% of symptoms on UPDRS scores [13]. In the absence of another diagnosis which can explain symptoms and based on United Kingdom Parkinson's Disease Society Brain Bank (UKPDSBB) criteria, she was diagnosed as having an early Parkinson disease (PD) [14]. The patient was initially treated with dopaminergic agonists therapy for 3 months without significant improvement. Tremor had partially decreased after 8 weeks of levodopa. In April 2014, the interrogation revealed sicca syndrome with xerophthalmia and xerostomia during the two previous years. The Schirmer test was clearly positive. Salivary gland scintigraphy showed hypofunction of the parotid glands. Cerebrospinal fluid (CSF) analysis revealed a pathologic IgG index (0.76) with a monoclonal band. Laboratory studies revealed the presence of antinuclear antibodies (1/80). The anti-SSA showed a high level. Tests for anti-DNA, anti-SSB, antiphospholipids, and thyroid antibodies were negative. Rheumatoid factor, total complement, and serum angiotensin convertase were normal. Cryoglobulinemia was not detected. Human immunodeficiency virus (HIV), herpes simplex virus, Lyme serologies, and the venereal disease research laboratory (VDRL)/*Treponema pallidum* hemagglutination assay (TPHA) test were all negative. Salivary gland biopsies showed a lymphocytic infiltration of grade 4 according to Chisholm's criteria. Accordingly, our patient fulfilled the pSS criteria, as proposed by Vitali and coworkers in 2002 [15]. The patient received high doses of intravenous methylprednisolone (1000 mg/day for 5 days), relayed by oral corticosteroid therapy. 12 weeks later, she showed a spectacular clinical improvement with remission of tremor and regression of rigidity. The patient, who reports periodically for consultation, keeps moderate rigidity.

## 3. Discussion

This report describes the case of a patient who had symptoms mimicking idiopathic PD in which pSS was later suspected and confirmed by further investigations. To the best of our knowledge, only 14 similar cases have been reported since the first publication of Visser and collaborators in 1993 [3]. Afterward, this association has been reported in isolated cases ([4–6, 8–11]; Table 1). Walker et al. in 1999 and Hassin-Baer et al. in 2007 described 3 cases, respectively [7, 12]. Analysis of these cases with our observation leads to the conclusion that Parkinsonism may be unilateral or bilateral, usually akineto-rigid nontremulous. It precedes the systemic signs in 8/15 cases. Women are more commonly subject to Parkinsonism, with an average age of 61 years, and are often dopa-resistant [4, 7, 9, 11]. In most cases, brain MRI shows hyperintensity on T2 and FLAIR in the white matter, in the striatum, or in the globus pallidus [3, 5–7, 10]. The MRI of our patient was normal, similar to some other published cases (Table 1). This reflects the lack of clinicopathological correlation between brain damage and neurological symptoms in the pSS. Response to corticosteroids was very variable. Some cases were corticoresistant. In the remaining cases, partial improvement can be obtained with corticosteroids unless with levodopa therapy [4]. Our patient was partially improved with levodopa and she got more improvement with corticosteroid therapy. This variable response to treatment raises many problems: First, can Parkinsonism be really secondary to SS? In this case, it seems to be an early sign of the disease. The hypothesis of an autoimmune process directed against the basal ganglia could be incriminated. The pathogenic role of anti-SSA and anti-SSB remains controversial [7]. In the literature, high titers of anti-beta2-glycoprotein IgG were found in the serum of 3 patients who had “pSS and Parkinsonism” association [12]. This autoantibody is strongly associated with anti-cardiolipin antibodies (aCL), antiphospholipid syndrome, and thromboembolic phenomena, but its role in the pathogenesis of the Parkinsonian disorder in SS is still unclear. These patients may present a subtype of SS patients in which aCL antibodies could directly attack the basal ganglia and be responsible of Parkinsonian symptoms. Finally, direct toxicity of T cells has also been raised after discovery of lymphocytic infiltration on histological sections of biopsied brain injury [14]. The young age of our patient and the improvement of neurological symptoms following corticosteroid therapy would suggest a causal link between Parkinsonism and pSS.

On the other hand, the hypothesis of a “defined PD and pSS” association remains possible: given that the evolution of Parkinsonian symptoms in our patient was progressive over 6 years with no systemic manifestation in the foreground, the signs were asymmetrical, the brain imaging was normal, and levodopa test was positive. The diagnosis of PD according to the UKPDSBB criteria could be considered, especially after short term levodopa responsiveness and the absence of atypical signs [14]. It should be kept in mind that these early onset PD forms exist in our country and would be linked to a mutation in the gene of parkin, PINK1, and LRRK2 [16]. It should be noted also that, over an 18-month follow-up period, the patient kept a good clinical outcome without the use of immunosuppressive agents. Thus, we can suggest that if it was a neuro-Sjögren Syndrome, evolution on steroids alone would not have been satisfactory.

Looking at PD, the question is whether there exists a shared immunopathogenesis between these two diseases one inflammatory and the other neurodegenerative. Currently, neuroinflammation in physiopathogenesis of PD is a subject of debate [17, 18]. Evidence of autoimmune involvement in PD was recently discussed [19]. Tumor necrosis factor-alpha (TNF-*α*), a proinflammatory cytokine involved in the SS as in other systemic diseases, was found with high titers in the blood, CSF, and striatum of patients with PD [20]. Recent autopsy results, genetics, and molecular imaging all suggest that inflammation plays a role in the neurodegenerative process [20]. These findings open a new therapeutic prospect for PD which has long been known as a neurodegenerative disease.

## 4. Conclusion

The literature reports only few cases of  “Parkinsonism and pSS.” A causal relationship has been discussed by some authors according to the response to treatment. Based on the analysis of our observation and review of the recent findings concerning the pathophysiology of PD, the association of “PD and pSS,” as two distinct diseases, cannot be excluded. The presence of a common neuroinflammatory process seems to be possible.

## Figures and Tables

**Table 1:** Cases of primary SS associated with Parkinsonism reported in the literature.

Authors	Sex/age	Diagnosis of SS before Parkinsonism	Initial clinical symptoms	MRI findings	Treatment	Development
Visser et al. [3]	F/55	No	Left hemiparkinsonian syndrome	Hyperintensities T2 in striatum and pallidum	Corticosteroids Azathioprine	No improvement

Nagao et al. [4]	F/79	No	Akineto rigide syndrome	Cortical atrophy and cortical hyper-T2 intensity	Levodopa Corticosteroids	No improvement Partial improvement

Creange et al. [5]	F/44	Unspecified	Postural instability	Hyper-T2 intensity of white matter	Corticosteroids Immunoglobulin	Transitory improvement

Mochizuki et al. [6]	H/50	No	Meningitis, akineto-rigide syndrome	Hyper-T2 intensity of white matter	Corticosteroids	Partial improvement

Walker et al. [7]	F/76	Yes	Akineto-rigide syndrome, cognitive disorders	Hyper-T2 intensity of white matter	Without corticosteroids Methotrexate	No improvement

Walker et al. [7]	F/63	Yes	Postural instability	Normal	Levodopa Corticosteroids	No improvement

Walker et al. [7]	F/53	No	Parkinsonian tremo akinéto rigide syndrome	Normal	Without treatment	No improvement

Nishimura et al. [8]	F/74	Yes	Postural instability	Diffuse hyper-T2 intensity		

Jafoui et al. [9]	F/39	No	Right hemiparkinsonian syndrome	Normal	Levodopa, bromocriptine Corticosteroids	No improvement Improvement

Gaundong Mbethe et al. [10]	F/67	No	Parkinsonian tremo akinéto rigide syndrome Headaches	Hyper-T2 intensity of white matter	Corticosteroids Cyclophosphamide	Improvement of tremor

Essaadouni et al. [11]	F/66	No	Akinéto-rigide syndrome	Frontal ischemic gaps and lenticular nuclei	Levodopa Piribedil	No improvement
					Corticosteroids	Improvement

Our case	F/46	No	Parkinsonian tremo akinéto rigide syndrome	Normal	Levodopa Corticosteroids	Partial improvement Improvement
